# Molecular mechanism of double Holliday junction dissolution

**DOI:** 10.1186/2045-3701-4-36

**Published:** 2014-07-09

**Authors:** Paolo Swuec, Alessandro Costa

**Affiliations:** 1 Clare Hall laboratories, Cancer Research U.K. London Research Institute, London EN6 3LD, UK

## Abstract

Processing of homologous recombination intermediates is tightly coordinated to ensure that chromosomal integrity is maintained and tumorigenesis avoided. Decatenation of double Holliday junctions, for example, is catalysed by two enzymes that work in tight coordination and belong to the same ‘dissolvasome’ complex. Within the dissolvasome, the RecQ-like BLM helicase provides the translocase function for Holliday junction migration, while the topoisomerase III alpha-RMI1 subcomplex works as a proficient DNA decatenase, together resulting in double-Holliday-junction unlinking. Here, we review the available architectural and biochemical knowledge on the dissolvasome machinery, with a focus on the structural interplay between its components.

## Introduction

RecQ helicases play a key role in genome stability maintenance. In humans, five distinct proteins containing a RecQ-like module are involved in a set of diverse nucleic acid transactions, including DNA replication, recombination and repair. Three of these proteins (the Werner syndrome helicase WRN, the Bloom’s syndrome protein BLM and RecQL4) are found mutated in rare genetic disorders, characterised by chromosomal aberrations that are in turn associated with cancer predisposition and premature aging [1]. Mutations in the BLM protein, for example, cause Bloom’s syndrome [2], whose hallmark is a pronounced increase in sister chromatid exchange [3].

### The dissolvasome complex collapses and unlinks a double Holliday junction

DNA double-strand breaks can be repaired by homologous recombination, whose key intermediate is the four-way Holliday junction. During meiosis, Holliday junctions are cut by endonucleases to generate crossovers that are key to the proper segregation of chromosomes [4,5]. In somatic cells crossovers may cause deleterious mutations and are suppressed by BLM [6]. In a process aided by topoisomerases [7-9], BLM catalyses the convergent migration of a double Holliday junction (dHJ), collapsing it into a hemicatenane [10]. This intermediate is unlinked by Topoisomerase IIIα (TopoIIIα) to generate non-crossover products [6]. BLM and TopoIIIα work in the context of a hetero-complex named the dissolvasome [10] that also includes the “RecQ-mediated instability factors”, RMI1 and RMI2 [11,12]. The interaction and concerted activities of the BLM and TopoIIIα complex is sufficient to drive dHJ dissolution [6] but RMI1 has been found to stimulate this reaction [13,14]. Although the molecular basis of dHJ dissolution remains unclear, a wealth of information is available on the structure and function of the isolated dissolvasome components and their subcomplexes.

### BLM catalyses the convergent migration of two Holliday junctions

**Figure 1:**
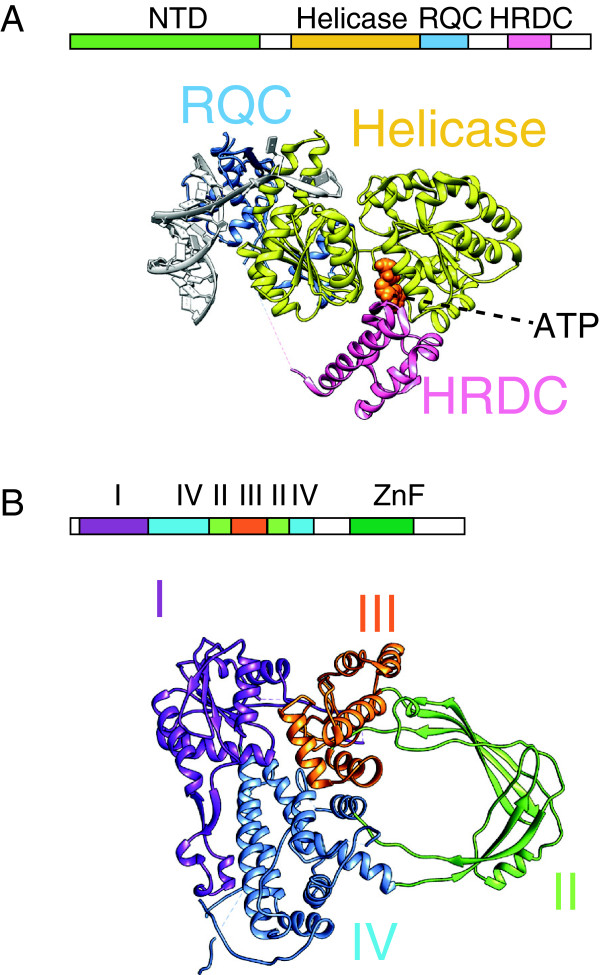
**Structure of the dissolvasome enzymatic components. (A)** Linear and three-dimensional structure of the human BLM helicase, in complex with a DNA and nucleotide substrate (PDB ID 4CGZ). **(B)** Linear and three-dimensional structure of human Topoisomerase IIIα (PDB ID 4CHT).

BLM (Sgs1 in yeast) provides the ATP-dependent motor function to convergently migrate a dHJ [15,16] and contains two separate domains (Figure 1A, [17]). The N-terminal region comprises a partially unstructured non-catalytic domain (NTD), which is the target of diverse post-translational modifications [18,19] and might contain a homo-oligomerisation module [20]. The BLM C-terminal domain contains the RecQ-like motor, belonging to the superfamily 2 of helicases, which are 3’ to 5’ single-stranded DNA translocases that can function as monomeric enzymes [21]. In RecQ proteins, a highly conserved helicase domain contains a bipartite active site for ATP binding and hydrolysis, where catalytic residues are contributed by two fused, neighbouring RecA-type modules [22]. The **R**ec**Q C**-terminal (**RQC**) domain contains a zinc finger motif and a duplex-DNA binding Winged-Helix subdomain [22], which provides the proteinaceous pin to split the two DNA strands at the fork nexus (Figure 1A, PDB entry 4CGZ and [23]). Unexpectedly, a minimal helicase module containing the RecA sandwich and the zinc finger domain but lacking the Winged-Helix domain has been recently described, which can unwind a fork substrate *in vitro*[24]. Lastly, the **H**elicase and **R**Nase**D C**-terminal (**HRDC**) domain is a separate, globular entity that confers substrate specificity to BLM [25,26], being required for Holliday junction dissolution or unwinding, but not for the unwinding of a simple DNA-fork substrate [27].

Various reports indicate that BLM can be found in distinct oligomeric states that co-exist in solution, ranging from monomers and dimers [28,29] to tetramers and hexamers [30]. The effect of substrate binding of the BLM oligomeric state is still a matter of debate and the functional implication of self-assembly is still unclear, however it might play a role in orienting multiple helicase motors for the convergent migration of two Holliday junctions during dissolution [17].

### Topoisomerase III alpha is structurally related to a type1A relaxase

**Figure 2:**
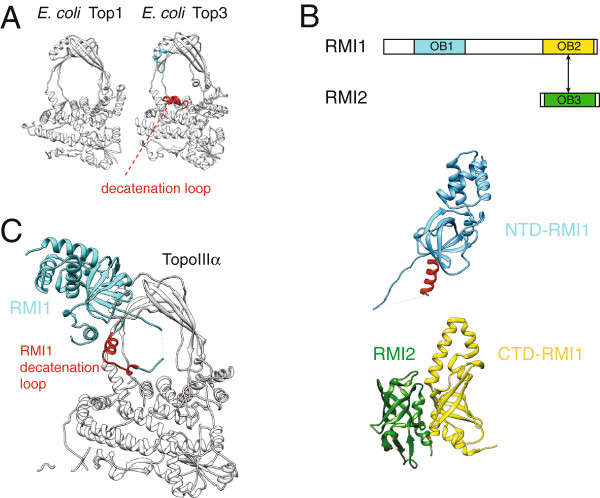
**Topoisomerase IIIα and RMI1 reconstitute a DNA decatenase. (A)** Structure of the *E. coli* TopoI relaxase (PDB ID 1ECL) and the *E. coli* TopoIII decatenase (PDB ID 1D6M). TopoIII contains specific insertions lining the pore of the topoisomerase toroid which are critical for efficient decatenation. **(B)** Linear and three dimensional structure of RMI1 and RMI2 (PDB IDs 3NBI and 4DAY). The decatenation loop of RMI1 is highlighted in red. **(C)** The N-terminal domain of RMI1 contributes to TopoIIIα a decatenation loop *in trans* (marked in red, PDB ID 4CGY).

The second key player in the dHJ dissolution reaction is TopoIIIα, which unlinks a hemicatenane intermediate during the final step of dissolution [10]. TopoIIIα belongs to the type1A class of topoisomerases, which are padlock-shaped enzymes that effect changes in DNA topology in an ATPase independent manner [31,32]. Type 1A topoisomerases are indeed markedly distinct from the ATP-dependent type-II topoisomerases and contain a 4-domain core (I-IV) that can bind, cleave and reseal single-stranded DNA substrates (Figure 1B, [33]). This process occurs through a transesterification reaction mediated by a nucleophilic tyrosine (Tyr337 in human TopoIIIα), thus creating a transient ‘DNA gate’ for nucleic-acid strand passage (between domains I and III) [34]. Type1A topoisomerases can be classified into two groups: *i)* relaxases (such as the *E. coli* TopoI), which efficiently remove negative supercoils from a covalently closed plasmid [35] and *ii)* decatenases (e.g. *E. coli* TopoIII), which can unlink catenated DNA molecules [36]. Although *E. coli* TopoI and TopoIII overall share the same fold, TopoIII contains small additional elements, as for example a short domain IV insertion (‘decatenation loop’), which lines the topoisomerase central cavity and is important for catenane unlinking (Figure 2A, [37,38]). Unexpectedly, TopoIIIα lacks the decatenation loop and appears structurally more similar to type1A relaxases than to decatenases [31], raising the question of how TopoIIIα-mediated hemi-catenane unlinking is achieved. Recent work indicates that RMI1 plays a key structural role in this process [31,39].

### RMI1 stabilises the open form of the TopoIIIα DNA gate

RMI1 stimulates the double-Holliday junction dissolution reaction and directly interacts with both the BLM helicase and TopoIIIα [13,14,40]. It is composed of an N-terminal helical bundle followed by an oligonucleotide/oligosaccharide binding (OB) domain, which is connected, via a poorly conserved linker, to a higher-eukaryote specific second OB scaffold (Figure 2B, [41,42]). The second OB fold in turn contacts the related RMI2 factor (absent in yeast), together providing a docking site for other genome stability maintenance machineries [43,44]. NTD RMI1 is sufficient to stimulate dHJ dissolution [42,45] and in particular acts in the later stages of this reaction [7,39]. In fact, while Rmi1 has no effect on the initial rate of Holliday junction migration, as shown in yeast, it has an important role in removing the last linkages between two recombining DNA molecules [7]. Further work in yeast has shown that Rmi1 acts by stabilising the open form of the Top3 DNA gate (orthologous to TopoIIIα), hence slowing down the kinetics of DNA relaxation while favouring decatenation [39]. Recent crystallographic studies on the human TopoIIIα-RMI1 sub-complex from Nico Thomäs group elucidate the molecular basis of the RMI1 stimulatory role in decatenation. The NTD RMI1 OB fold docks onto TopoIIIα domain II and contributes an insertion loop that lines the topoisomerase central cavity, while also reaching out to domain III at the DNA gate (Figure 2C, [31]). Close inspection of the TopoIIIα-RMI1 complex reveals that the RMI1 insertion loop indeed contributes topoisomerase-interacting elements in regions where TopoIIIα diverges from the *E. coli* TopoIII decatenase (Figure 2D, see previous paragraph). Hence, RMI1 appears to donate a DNA decatenation loop to TopoIIIα in *trans*, which could be sufficient to turn a relaxase into a decatenase. Although the crystal structure of TopoIIIα-RMI1 was solved in a closed configuration, the insertion loop appears poised to push the gate open, locking the topoisomerase in a covalently-attached, open, DNA-bound form that would promote DNA decatenation. Consistent with this model, a scrambled insertion-loop mutant, which does not interfere with complex formation, fails to stimulate dHJ dissolution, reverting yeast Top3 back to a modest decatenase [31].

### BLM plays a structural role in modulating strand passage

How BLM structurally and functionally interacts with the TopoIIIα–RMI1 sub-complex is still poorly understood. It is known that BLM contacts NTD RMI1 [13,14,45] while TopoIIIα interacts with the NTD of BLM/Sgs1 [46,47]. In *Drosophila*, which appears to lack RMI1, a fly-specific C-terminal insertion in TopoIIIα that contacts BLM at an unknown site, is essential for dissolution and might functionally substitute RMI1 [9]. It remains to be established whether the RecQ and Top1A catalytic domains ever come in direct contact during dissolution and whether any allosteric communication occurs between the two modules.

Similarly unclear is the nature of the BLM-TopoIIIα catalytic interplay [48]. Work in *Drosophila*, for example, suggests that BLM and TopoIIIα could coordinate their activities to achieve branch migration [8]. *In vitro* studies on this system indicate that, while the isolated BLM is incapable of migrating a topologically constrained dHJ, vigorous migration is observed when *Drosophila* Top1 is added to the reaction mixture, although branches pause before merging into a hemicatenane. When BLM is instead assayed with TopoIIIα, the reaction goes to completion without any pausing, indicating that dissolution is indeed a highly processive reaction, and hinting at a possible catalytic interplay between the two enzymes [8].

Work in yeast, however, indicates that Sgs1/BLM has a role in modulating strand passage by TopoIIIα and RMI1, which does not depend on the catalytic activity of the helicase module [39]. In a catenation assay on plasmids containing a preformed DNA bubble, Sgs1-Top3-Rmi1 appears proficient in converting individual DNA molecules into multimeric catenanes, irrespective of the presence of a wild type or a catalytically-dead mutant of Sgs1 [39]. These results indicate that Sgs1/BLM has a structural, rather than catalytic, role in promoting strand passage in the dissolvasome. It remains to be determined whether Sgs1/BLM binding acts by repositioning the decatenation loop insertion in RMI1 or rather contacts the topoisomerase gate directly, hence controlling the TopoIIIα opening/closure state.

## Concluding remarks

Recent advances shed light on the role of isolated dissolvasome components in the convergent migration of dHJ junctions and hemi-catenane unlinking. Whether BLM and TopoIIIα-RMI1 allosterically influence their reciprocal catalytic activities still remains unclear. A comprehensive view of the molecular basis of dHJ dissolution will be likely achieved once the high-resolution structure of the full dissolvasome assembly is determined.

## Competing interests

The authors declare that they have no competing interests.

## Authors' contributions

PS and AC wrote the manuscript. Both authors read and approved the final manuscript.
